# Characterization of mycotoxins and microbial community in whole-plant corn ensiled in different silo types during aerobic exposure

**DOI:** 10.3389/fmicb.2023.1136022

**Published:** 2023-03-27

**Authors:** Guang-hao Xia, Yuan Huang, Chang-rong Wu, Ming-zhu Zhang, Hai-yan Yin, Feng Yang, Chao Chen, Jun Hao

**Affiliations:** ^1^College of Animal Science, Guizhou University, Guiyang, China; ^2^Guizhou Grassland Technology Extending Station, Guiyang, China; ^3^Key Laboratory of Animal Genetics, Breeding and Reproduction in the Plateau Mountainous Region, Ministry of Education, Guizhou University, Guiyang, China

**Keywords:** whole-plant corn silage, aerobic exposure, microbial community, mycotoxins, fermentation

## Abstract

Silage can be contaminated with mycotoxins and accidental fungi after aerobic exposure. The study assessed the effects of bunker silos (BS), round bales (RB), and silage bags (SB) on the nutritional characteristics, fermentation quality, aerobic stability, mycotoxin levels and microbial communities of whole-plant corn silage (WPCS). After 90 days of fermentation, silages were opened and sampled at 0, 1, 3, 5, 7, and 9 days of exposure. SB group conserved higher lactic acid and dry matter contents and a lower pH value than other groups after 9 days of exposure (*p* < 0.05). The SB group showed the longest aerobic stability (202 h) among all silages (*p* < 0.05). The concentrations of aflatoxin B1, trichothecenes and fumonisin B1 were significantly lower in SB after 9 days of exposure (*p* < 0.05). *Acetobacter* became the dominant bacteria in BS and RB groups after 5 days of exposure. However, *Lactobacillus* still dominated the bacterial community in SB group. *Acetobacter* was positively correlated with pH, acetic acid content, and ammonia-N content (*p* < 0.05). *Lactobacillus* was positively correlated with *Kazachstania* and *Candida* abundances (*p* < 0.01) but negatively correlated with *Fusarium* abundance (*p* < 0.05). Considering the feed value and food safety of silage in the feeding process, silage bags are recommended for WPCS according to the observed nutritional quality, fermentation index and mycotoxin content.

## 1. Introduction

Food safety has received increasing international attention in public regulation, private supply chain coordination, and international trade in recent years (Unnevehr, 2015). The World Health Organization (WHO) estimated that there are approximately 600 million illnesses and 420,000 deaths worldwide caused by 31 foodborne hazards annually (World Health Organization, 2015). Livestock products such as meat, milk, and eggs represent a considerable source of animal protein for human food (Cai et al., 2021). However, these products are easily spoiled by many factors, such as zoonotic diseases, mycotoxins and undesired microorganisms. To prevent the negative effects associated with livestock products, it is suggested that feed safety should be considered a prerequisite in developing a farm-to-fork food safety program for animal source foods (FAO, 2019). Silage plays an important role in the global agricultural and agri-food industries, ensuring a constantly nutritious supply for ruminants, especially whole-plant corn silage (WPCS), which is used in ruminant feeding as an important source of a high energy content and digestible fiber (Khan et al., 2015; Gheller et al., 2021). However, silage can be contaminated with biological and chemical hazards from inherent mycotoxins and accidental fungi when there is a lack of standardized manufacturing and processing, storage or transport (Ghilardelli et al., 2022). This contamination is worse during the aerobic exposure period than during other stages. Da Silva et al. (2015) reported that increases in temperature and pH could lead to silage decay significantly after silage was exposed to air. Several studies have shown that ensiled forage, particularly the superficial silage in bunker silos (BS), usually accumulates mycotoxins such as aflatoxin B1 (AFB1), trichothecenes (T-2), fumonisin B1 (FB1), zearalenone (ZEN), deoxynivalenol (DON), and many other fungal secondary metabolites under aerobic exposure conditions (Ogunade et al., 2018). It was also found that intake of mycotoxins destroys the structure and function of the animal intestinal tract, damages the immune and antioxidant systems, causes disorders of intestinal metabolism, and eventually impairs the health of ruminants (Chen et al., 2022). Furthermore, there is a potential possibility that mycotoxins would accumulate in livestock products such as meat, milk, eggs, and blood products. Intake of livestock products with mycotoxins causes destructive effects on humans including altered genome expression and kidney diseases, diminished reproductive system activity, the intestinal tract disruption, and the development of cancer-causing cells in the body (Luo et al., 2018).

Bunker silos (BS), round bales (RB) and silage bags (SB) are becoming common ways to store silage. A lack of scientific ensiling management usually results in poor chemical composition and excessive butyric acid contents (Liu et al., 2019). Feeding WPCS from BS has the advantage of being more efficient than feeding WPCS from RB and SB. The plastic consumption per gram of crop in BS was calculated to be more than 5 times lower than that in SB (Randby et al., 2020). However, there have been opposite conclusions considering the nutritional quality and feeding value of silages. Muck et al. (2015) found that silage quality was worse in BS than in SB and tower silo. Randby and Bakken (2021) also reported a similar phenomenon in which the sum of dry matter (DM) lost by crop respiration, effluent runoff, anaerobic fermentation, aerobic deterioration and gaseous losses was significantly higher in BS than in RB.

However, most previous studies have focused on comparing different ensiling methods on the commercial value of WPCS during fermentation, ignoring its quality and safety to livestock in the process of utilization with air exposure. Hence, the objectives of this study were to investigate the effect of BS, RB and SB on the chemical characteristics, fermentation quality, aerobic stability, microbial community, and mycotoxin contents of WPCS during an aerobic exposure period (0, 1, 3, 5, 7, and 9 days). Furthermore, the results will be helpful to determine an optimal ensiling method to limit silage degradation and to reduce the impact of poor silage quality on ruminants and human health.

## 2. Materials and methods

### 2.1. Silage preparation

Row whole-plant corn (Qingfeng 4) was obtained from a commercial plant base located in Liupanshui City, Guizhou Province (104.83 °E, 26.60 °N) on September 30, 2020 during the dough stage. The plant material was chopped to a theoretical cut length of 1–2 cm by a precision chopper. After mixing thoroughly, the chopped whole-plant corn was divided into three parts for: (i) ensiling in BS (7 m × 24 m with three 3.2 m high walls, and a capacity of 280 tones fresh crop weight), (ii) ensiling in RB (bales were immediately wrapped with 8 layers of 0.75 m wide and 0.025 mm thick black plastic film, dimensions: 0.8 m diameter × 0.6 m length, with a maximal capacity of 190 kg fresh crop weight), and (iii) ensiling in SB (dimensions: 0.5 m wide × 0.6 m height × 1.0 m length, and with a maximal capacity of 200 kg fresh crop weight). After 90 days of fermentation, silages were opened for 9 days of aerobic exposure. The density of the BS ranged from 496 to 504 kg/m^3^, while that of the RB and SB groups ranged from 592 to 604 kg/m^3^ and 644 to 655 kg/m^3^, respectively. The ambient temperature ranged from 0 to 7.5°C. Samples (about 1 kg each) from the middle of silages were taken at the same six time points (0, 1, 3, 5, 7, and 9 days of aerobic exposure) during the unloading of the BS, RB and SB, respectively. For BS, samples were collected from 3 silos as three replicates at 6 different sampling times. For RB and SB, totally 36 silos were taken for ensiling, all silos were opened and samples were collected from 3 silos of each treatment at one sampling time. In total, the study comprised 3 BS, 18 RB, and 18 SB. The samples (3 treatments × 6 time points × 3 replicates) were used to determine the chemical composition, fermentation quality, aerobic stability, microbial community and mycotoxin contents. One part of silage was sampled in 50 mL cryogenic vials and stored in a −80°C freezer for microbial community analysis, and another part was stored in a −20°C freezer for chemical composition, fermentation quality, and mycotoxin content analysis.

### 2.2. Chemical, fermentation and aerobic stability analyses

20 g of silage samples were combined with 180 mL of distilled water and stored in a 4°C refrigerator for 1 day, then four layers of cheese cloth were used to filter the silage, for the determination of the fermentation profile. The pH was measured using a pH meter (PHSJ-3F, CANY, Shanghai, China). Concentrations of lactic acid, acetic acid, propionic acid and butyric acid were determined by high-performance liquid chromatography, according to the methods described by Wu et al. (2022). The ammonia-N content was measured using phenol-hypochlorite colorimetry (Broderick and Kang, 1980).

WPCS was dried at 65°C for 48 h in a dry oven to measure the dry matter (DM) content. Then grounded into powder for total nitrogen, crude protein (CP), water-soluble carbohydrate (WSC), neutral detergent fiber (NDF) and acid detergent fiber (ADF) analyses. The CP content was calculated *via* the total nitrogen content multiplied by 6.25, and the total nitrogen was analyzed by a Kjeldahl nitrogen analyzer (Kjeltec 8400 Analyzer; Foss, Sweden). According to the methods described by Turula et al. (2010), WSC was determined by colorimetric after-reaction with anthrone reagent. The NDF and ADF were measured based on Van Soest procedures, a heat stable alpha amylase was used in the NDF procedure, the results were expressed on a DM basis including residual ash. Aerobic stability was measured after 90 days of ensiling. About 3 kg of silages from each silo were taken and mixed thoroughly, then put into separate new plastic silos (capacity 10 L) without compaction and uncovered. The geometric-center of the silage masses and the ambient temperature were recorded with sensors every 2 h. The aerobic stability was determined based on the time when the temperature of silage exposed to air exceeded the ambient temperature by 2°C (Ranjit and Kung, 2000).

### 2.3. Analyses of the microbial community

WPCS (20 g) was homogenized with distilled water (180 mL), and subsequently filtered through two layers of medical gauze and centrifuged for 5 min at 8,000 *g*/min to collect microorganism cells. The total genomic DNA was extracted *via* the HiPure Soil Kit (QIAGEN, Inc., Venlo, Netherlands), then the purity, concentration, and integrity of the isolated DNA samples were determined by agarose gel electrophoresis. Thereafter, the 16S rRNA V5–V7 regions of genomic DNA was amplified *via* Pyrobest DNA Polymerase (TaKaRa, DR500A) with the universal primers of 799F (AACMGGATTAGATACCCKG) and 1193R (ACGTCATCCCCACCTTCC). ITS genes of regions (ITS1_other) were amplified using the specific primers ITS1-F (CTTGGTCATTTAGAGGAAGTAA) and ITS2 (GCTGCGTTCTTCATCGATGC) with Barcode. AMPure XP Beads (Beckman Coulter, Indianapolis, IN, United States) was adopted for the purification of the polymerase chain reaction (PCR) products, and the ABI StepOnePlus Real-Time PCR System (Life Technologies, United States) was used for quantification (Toju et al., 2012). Following the generate sequencing libraries according to the manufacturer’s instructions, the library quality was performed on the Illumina HiSeq 2500 platform by Gene Denovo Biotechnology Co., Ltd. (Guangzhou, China). After high-throughput sequencing and the filtration of chimera and low-quality sequences, the bioinformatics analyses of the microbial community were mainly performed using the QIIME (Version 2.15.3) and R software (Version 4.0.0).

### 2.4. Mycotoxin analyses

Mycotoxins were extracted simultaneously from 2 g of a dried silage sample using a 40 mL centrifuge tube with 20 mL of an acetonitrile: water solution (80:20 v/v). After the samples were horizontally shaken for 40 min, 1.0 g of NaCl and 2.0 g of anhydrous magnesium sulfate was added to obtain phase separation. After shaking for another 1 min and centrifugation at 6,000 *g*/min for 5 min, the upper acetonitrile phase was recovered. The mixture was evaporated to dryness under a nitrogen steam at 40°C and re-dissolved in methanol: formate in water solution (10:90, v/v), then the final extract was filtered through a filter (Millipore Corporation, Bedford, United States; HV 0.45 μm). A 20 μL sample was injected into the HPLC with MS/MS system according to the methods described by Gallo et al. (2010).

### 2.5. Statistical analysis

Results are reported as the mean and the standard error of the mean (SEM). The data related to fermentation quality, chemical composition, mycotoxin levels and alpha diversity were subjected to two-way analysis of variance. Aerobic stability of WPCS after ensiling were subjected to one-way analysis of variance. When there were significant differences (*p* < 0.05), the group means were further compared with Duncan’s multiple range tests. The statistical analyses were performed using SPSS 26.0 (SPSS, Chicago, IL, United States). Alpha diversity metrics (Chao1, Shannon and Good’s coverage) were calculated with QIIME software (Version 2.15.3). Sample ordination based on the beta diversity was examined using the principal coordinate’s analysis (PCoA). The relative abundances of microbial communities at the phylum and genus levels were also analyzed. The resultant correlation matrix was analyzed by “corrplot” in R language (method = Spearman).

## 3. Results

### 3.1. Silage characteristics and aerobic stability of WPCS during aerobic exposure

The chemical composition of the WPCS was shown in Table 1. The content of DM first increased and then decreased significantly over time (*p* < 0.05), and the highest DM content was observed at 3–5 days. The content of DM in each group was in the order of SB > RB > BS at any stage of aerobic exposure. The WSC content decreased after 9 days of aerobic exposure (*p* < 0.05), and the WSC content in the RB and BS groups was lower than that in the SB group. The CP content of the three groups showed a similar decreasing trend with increasing aerobic exposure duration. Compared to the RB and BS groups, the SB group retained the highest CP content after 9 days of aerobic exposure. There were significant increases in NDF and ADF contents during the exposure period in all groups (*p* < 0.05). The BS group had higher NDF and ADF contents than the other groups after 5 days of aerobic exposure. It was also found that the aerobic exposure days, ensiling methods, and their interaction significantly affected the contents of DM, CP, NDF, and ADF in the WPCS (*p* < 0.001).

**Table 1:** Effect of different ensiling methods on chemical compositions of WPCS during aerobic exposure.

Item	Ensiling methods	Aerobic exposure days						SEM	*p*-value		
		0	1	3	5	7	9		M	D	M × D
DM (g/kg FM)	BS	209.0Ccd	211.5Cbc	218.3Ca	214.8Cab	204.5Cd	183.9Ce	1.61	***	***	***
	RB	232.6Bbc	237.2Bab	238.7Ba	231.0Bcd	225.3Bd	210.6Be				
	SB	245.4Ac	246.6Abc	249.5Aab	252.6Aa	241.4Ad	231.3Ae				
WSC (g/kg DM)	BS	15.0Ca	13.6Cb	13.0Cb	13.2Bb	11.6Cc	11.2Bc	0.616	***	***	NS
	RB	17.0Ba	15.4Bab	14.5Babc	12.5Bbc	13.2Bc	12.7ABc				
	SB	22.3Aa	21.4Aab	20.4Ab	18.2Ac	17.5Ac	16.5Ac				
CP (g/kg DM)	BS	75.6Aa	74.8Aab	73.1Ab	67.7Ac	60.4Bd	44.4Ce	0.626	***	***	***
	RB	76.1Aa	73.7Bb	73.5Ab	67.2Ac	65.3Ac	59.9Bd				
	SB	75.4Aa	71.1Cb	69.3Bc	66.9Ad	64.6Ae	63.8Ae				
ADF (g/kg DM)	BS	183.1Bd	197.6Ac	205.9Ac	236.3Ab	239.6Ab	250.4Aa	2.306	***	***	***
	RB	192.7Ad	203.1Ac	206.5Abc	209.5Bb	225.5Ba	228.5Ba				
	SB	172.4Cc	182.7Bb	185.9Bb	201.4Ca	206.5Ca	207.8Ca				
NDF (g/kg DM)	BS	411.7Af	431.9Ae	441.8Ad	462Ac	474.8Ab	510.4Aa	4.093	***	***	***
	RB	415.6Ac	416.4Bc	431.6Bbc	434.1Cbc	450.7Cb	490.3Ba				
	SB	392.0Ad	415.9Bc	421.1Cc	455.1Bb	466.5Ba	471.1Ca				

FM, fresh matter; DM, dry matter; CP, crude protein; NDF, neutral detergent fiber; ADF, acid detergent fiber; WSC, water soluble carbohydrates. BS, bunker silo; RB, round bale; SB, silage bag; M, ensiling methods; D, aerobic exposure days; M × D, interaction of ensiling methods and aerobic exposure days. Means with different letters in the same row (a–f) or column (A–C) differ (*p* < 0.05). ***p < 0.001; NS, not significant; SEM, error of the means.

As shown in Table 2, the pH of the WPCS was significantly affected by the prolonged aerobic exposure duration (*p* < 0.001). The pH value of SB group ranged from 3.78 to 4.01 from d 0 to d 9, while BS and RB group had increased over 4.20 at d 3 and d 5, respectively. Although the contents of LA decreased with prolonged aerobic exposure times, the SB group had a higher concentration than the other groups. Lower PA and AA contents were also found in the SB group (*p* < 0.05), and PA was not detected until day 7. Furthermore, BA was not detected in the RB and SB groups. The content of ammonia-N in all groups were increased after 9 days of aerobic exposure (*p* < 0.05). The lowest ammonia-N content was found in the SB group on all days of aerobic exposure.

**Table 2:** Effect of different ensiling methods on fermentation quality of WPCS during aerobic exposure.

Item	Ensiling methods	Aerobic exposure days						SEM	*p*-value		
		0	1	3	5	7	9		M	D	M × D
pH	BS	4.21Ac	3.90Ad	4.31Ac	4.37Abc	4.52Aab	4.58Aa	0.033	***	***	NS
	RB	4.10Bc	3.79Bd	4.19Bb	4.21Bb	4.25Ba	4.27Ba				
	SB	3.78Cc	3.49Cd	3.91Cb	3.94Cb	3.96Cab	4.01Ca				
LA (g/kg DM)	BS	23.04Ba	20.93Cb	18.84Cc	17.54Bcd	16.22Bd	14.08Be	0.524	***	***	NS
	RB	22.19Ab	24.45Ba	21.33Bb	18.14Bc	17.50Bc	15.93Bd				
	SB	31.04Aa	29.25Ab	28.97Ab	27.10Ac	26.06Acd	24.50Ad				
AA (g/kg DM)	BS	12.51Ba	13.26Ba	10.00Bb	8.63Bc	7.88Bcd	7.43Bd	0.431	***	***	*
	RB	18.99Aa	19.22Aa	17.20Aa	14.02Ab	14.38Ab	13.02Ab				
	SB	11.51Bb	13.17Ba	10.81Bb	9.81Bc	8.78Bd	6.56Be				
PA (g/kg DM)	BS	4.77a	3.68c	3.85bc	4.22abc	4.05Bbc	4.34Aab	0.124	***	***	***
	RB	3.29c	3.33c	3.78b	3.54bc	4.50Aa	4.40Aa				
	SB	ND	ND	ND	ND	0.16C	0.28B				
BA (g/kg DM)	BS	0.80c	1.52ab	1.67ab	1.77ab	1.88ab	2.08a	0.079	***	***	***
	RB	ND	ND	ND	ND	ND	ND				
	SB	ND	ND	ND	ND	ND	ND				
Ammonia-N (g/kg total N)	BS	24.72Ad	27.70Ac	27.87Bc	31.47Ab	31.10Ab	35.55Aa	0.166	***	***	***
	RB	23.27Be	25.95 Bd	29.16Ac	28.98Bc	31.39Ab	33.44Ba				
	SB	21.02Cd	22.71Cc	27.60Bb	27.82Cb	28.10Bb	30.81Ca				

DM, dry matter; LA, lactic acid; AA, acetic acid; PA, propionic acid; BA, butyric acid; Ammonia-N, ammonia nitrogen; BS, bunker silo; RB, round bale; SB, silage bag; M, ensiling methods; D, aerobic exposure days; M × D, interaction of ensiling methods and aerobic exposure days. Means with different letters in the same row (a–f) or column (A–C) differ (*p* < 0.05). ***p < 0.001; *p < 0.05; NS, not significant; ND, not detected; “–,” default. SEM, error of the means.

**Figure 1:**
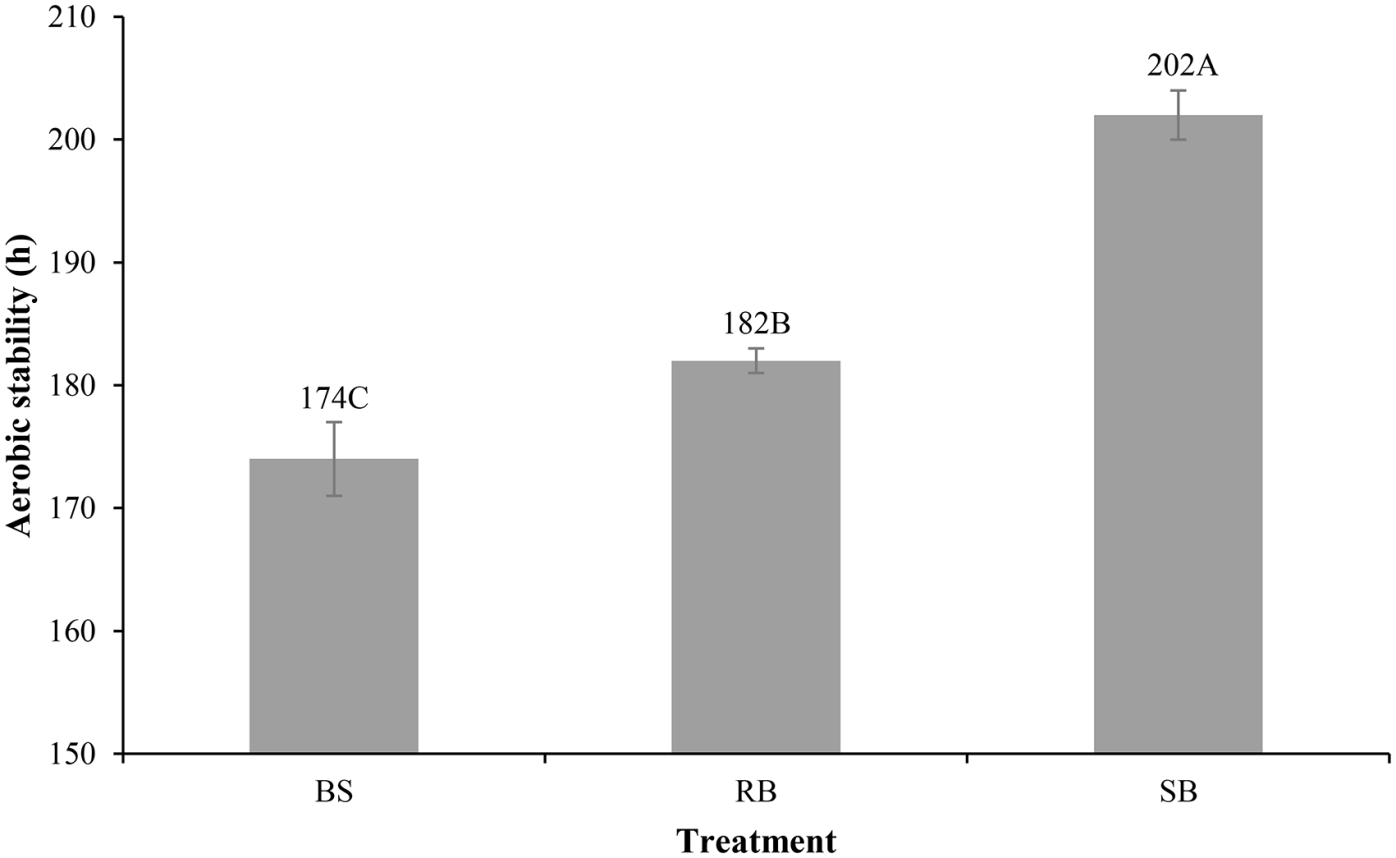
Effect of different ensiling methods on the aerobic stability (h) of WPCS after 90 days of ensiling (SEM = 1.247, *p* < 0.001). Vertical bars are the standard errors of the means, bars with different letters differ (*p* < 0.05). Treatment: BS, Bunker silo; RB, Round bale; SB, Silage bag.

The different ensiling methods significantly (*p* < 0.001) influenced the aerobic stability of the WPCS (Figure 1). The BS group spoiled within 174 h, RB group was stable for 182 h, which was (*p* > 0.05) longer than BS group (8 h). The SB group showed the strongest aerobic stability (202 h) among all silage.

### 3.2. Mycotoxin levels in WPCS during aerobic exposure

The results of the mycotoxin levels are summarized in Table 3. The AFB1, ZEN, T-2, DON and FB1 concentrations showed a significant interaction between ensiling methods and aerobic exposure days (*p* < 0.001). Overall, the concentrations of mycotoxins continually increased with increasing aerobic exposure (*p* < 0.05), and the BS group had higher concentrations than the RB and SB groups after 9 days of exposure (*p* < 0.05). AFB1, T-2 and FB1 concentrations were significantly lower in the SB group than in the other groups (*p* < 0.05), while the contamination levels of ZEN and DON did not show significant differences between the RB and SB groups after 9 days of oxygen exposure (*p* > 0.05).

**Table 3:** Effect of different ensiling methods on mycotoxins concentration of WPCS during aerobic exposure.

Mycotoxins	Ensiling methods	Aerobic exposure days						SEM	*p*-value		
		0	1	3	5	7	9		M	D	M × D
AFB1 (μg/Kg DM)	BS	5.83Ad	6.51Ad	7.83Ac	13.15Ab	14.21Ab	16.34Aa	0.392	***	***	***
	RB	5.71Ad	5.87Bd	6.73ABd	8.01Bc	10.24Bb	13.91Ba				
	SB	4.68Ad	5.49Ccd	5.65Bcd	6.14Cc	7.82Cb	9.66Ca				
ZEN (μg/Kg DM)	BS	5.56Ae	11.40Ad	13.21Acd	15.82Ac	25.59Ab	35.44Aa	0.705	***	***	***
	RB	5.49Ad	5.72Bd	6.45Bcd	8.02Cc	12.40Bb	18.02Ba				
	SB	4.50Bd	5.22Bd	6.60Bc	11.83Bb	13.78Ba	14.36Ba				
T-2 (μg/Kg DM)	BS	20.24Ad	24.46Ad	30.48Ac	34.18Ac	47.85Ab	64.26Aa	1.235	***	***	***
	RB	11.85Bf	15.79Be	19.36Bd	28.72Cc	37.47Bb	49.55Ba				
	SB	12.40Bd	16.81Bc	18.51Bc	31.66Bb	35.10Bb	40.03Ca				
DON (μg/Kg DM)	BS	126.84Ae	133.87Ad	144.79Ac	149.54Ac	167.88Ab	188.66Aa	2.047	***	***	***
	RB	123.59Ac	129.80Ac	130.60Bc	141.70Bb	153.04Ba	159.36Ba				
	SB	122.37Ac	126.52Ac	133.50Bb	155.50Aa	157.79Ba	162.19Ba				
FB1 (μg/Kg DM)	BS	25.48Af	30.78Ae	34.04Ad	38.20Ac	46.40Ab	59.09Aa	0.933	***	***	***
	RB	21.90Cf	24.92Be	31.61Ad	38.81Ac	42.36Bb	50.26Ba				
	SB	23.49Ba	23.30Ba	26.61Ba	35.17Bc	40.03Cb	44.53Ca				

AFB1, aflatoxin B1; ZEN, zearalenone; T-2, T-2 toxin; DON, deoxynivalenol; FB1, fumonisin B1. BS, bunker silo; RB, round bale; SB, silage bag; M, ensiling methods; D, aerobic exposure days; M × D, interaction of ensiling methods and aerobic exposure days. Means with different letters in the same row (a–f) or column (A–C) differ (*p* < 0.05). ***p < 0.001; SEM, error of the means.

### 3.3. Microbial community diversity in the WPCS during aerobic exposure

The Good’s coverage values were greater than 99%, indicating that the sampling depth adequately captured most of the bacterial and fungal communities. The alpha diversities of the WPCS were evaluated through Shannon and Chao1 indexes (Table 4). The ensiling methods significantly affected the alpha diversities of the microbial community (*p* < 0.001). The RB group had lower Shannon and Chao1 indexes than the BS and SB groups in the bacterial community (*p* < 0.05). However, the SB groups maintained lower Shannon and Chao1 indexes in the fungal community (*p* < 0.05).

**Table 4:** Alpha diversity of microbial community during aerobic exposure.

Items		Ensiling methods	Aerobic exposure days						Mean	SEM	*p*-value		
			0	1	3	5	7	9			M	D	M × D
Bacterial community	Shannon	BS	4.26	4.06	4.17	3.60	3.57	3.95	3.93A	0.347	***	NS	NS
		RB	2.98	2.57	2.60	2.32	3.21	2.36	2.67B				
		SB	3.80	3.97	3.89	3.96	4.09	4.00	3.95A				
		Mean	3.68	3.53	3.55	3.29	3.62	3.44					
	Chao1	BS	563	588	579	601	569	598	583A	32.204	***	NS	NS
		RB	455	355	414	334	446	421	404C				
		SB	466	456	474	469	427	398	448B				
		Mean	495	466	489	468	480	472					
Fungal community	Shannon	BS	3.06	3.74	2.72	4.41	4.26	3.75	3.66A	0.35	***	NS	*
		RB	2.40	2.44	2.75	2.38	1.86	1.26	2.18B				
		SB	2.09	1.65	2.19	2.34	1.90	2.19	2.06B				
		Mean	2.52	2.61	2.55	3.04	2.67	2.40					
	Chao1	BS	169	229	210	243	258	213	220A	25.780	***	NS	NS
		RB	165	251	213	189	188	158	194A				
		SB	162	143	193	173	167	111	158B				
		Mean	165	207	205	202	205	161					

BS, bunker silo; RB, round bale; SB, silage bag; M, ensiling methods; D, aerobic exposure days; M × D, interaction of ensiling methods and aerobic exposure days. Means with different letters in the same column (A–C) differ (*p* < 0.05). ***p < 0.001; *p < 0.05; NS, not significant; SEM, error of the means.

**Figure 2:**
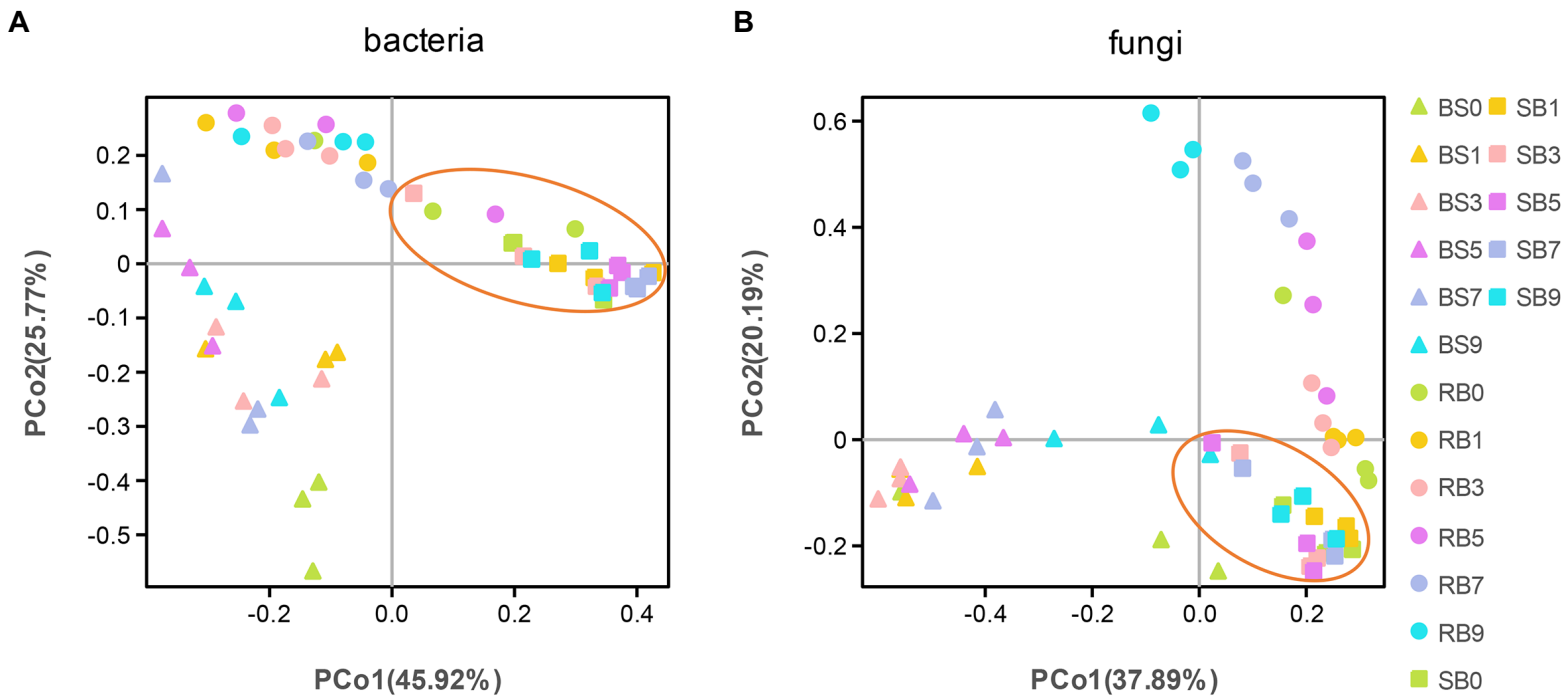
**(A, B)** Principal Coordinate Analysis (PCoA) of the bacterial and fungal community of the WPCS during different aerobic period. BS, Bunker silo; RB, Round bale; SB, silage bag. 0, 1, 3, 5, 7, 9: 0, 1, 3, 5, 7, 9 days of aerobic exposure, respectively.

To analyze the distribution and structure of bacterial and fungal communities in WPCS samples at different aerobic times, principal coordinates analysis (PCoA) based on Bray–Curtis distance at the OTU level was conducted (Figure 2). Good separation and differences in microbial communities were observed between different ensiling methods, and the microbial community of SB group showed less variation during aerobic exposure stages.

### 3.4. Bacterial community dynamics in the WPCS during aerobic exposure

**Figure 3:**
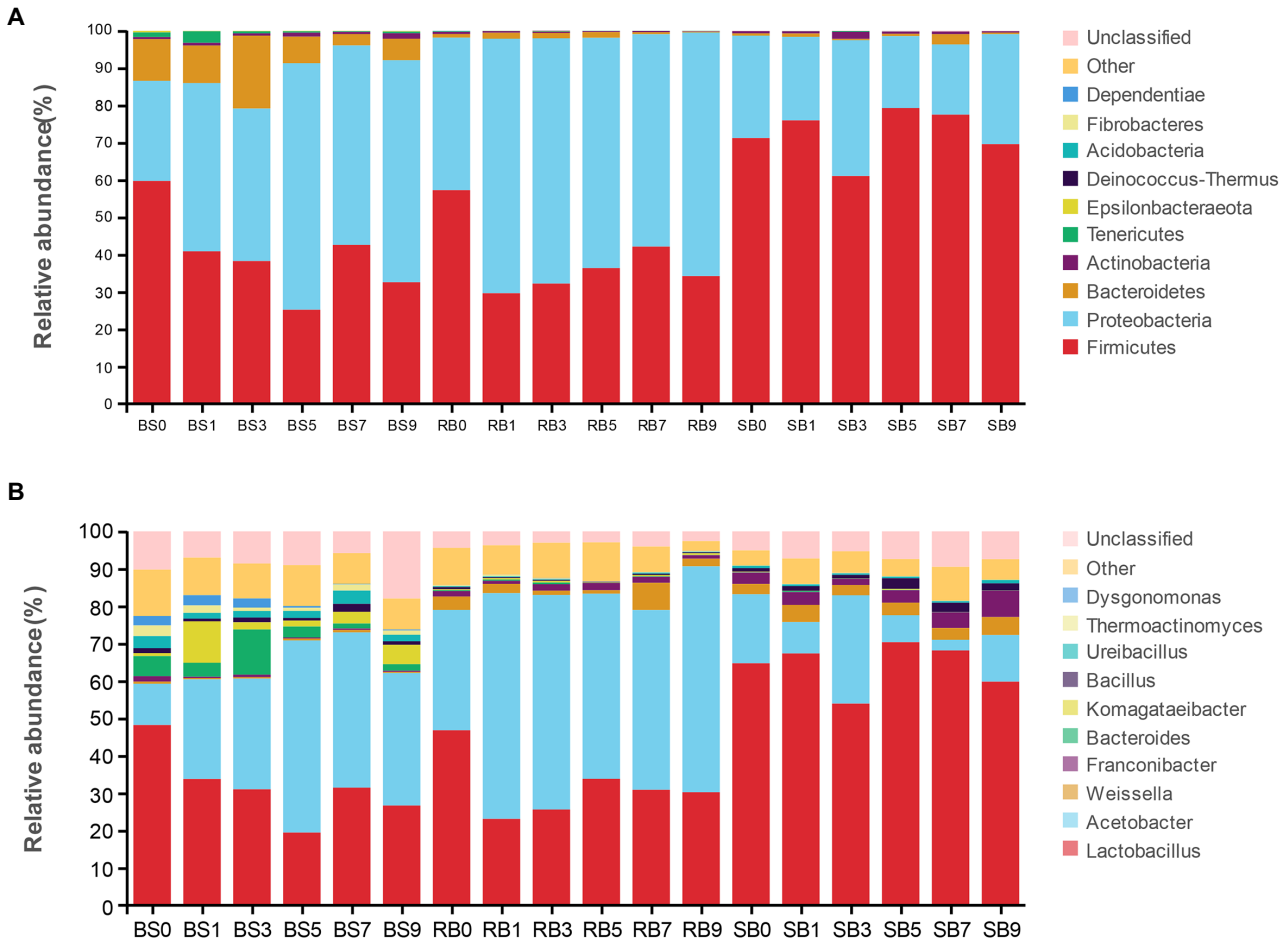
The dynamic bacterial community of the WPCS during aerobic exposure. The bacterial communities were shown at the phylum level **(A)** and the genus level **(B)**. BS, Bunker silo; RB, Round bale; SB, silage bag. 0, 1, 3, 5, 7, 9: 0, 1, 3, 5, 7, 9 days of aerobic exposure, respectively.

Alterations in the bacterial community at the phylum level after aerobic exposure are presented in Figure 3A. Overall, Proteobacteria and Firmicutes were the dominant phyla in all of the samples, covering more than 80% of the total sequences observed. The abundance of Proteobacteria increased dramatically in the BS and RB groups within 1 day of aerobic exposure, and Proteobacteria then became the most abundant phylum with prolonged exposure days. However, Firmicutes remained the most abundant phylum in the SB group despite exposure to the air.

As shown in Figure 3B, the relative abundances of the bacterial community were further analyzed at the genus level. *Lactobacillus* and *Acetobacter* were the most dominant genera in all treatments. The abundance of the genus *Lactobacillus* in the BS group decreased significantly with increasing exposure time, while the relative abundance of *Acetobacter* increased. The relative abundance of *Acetobacter* in the RB group increased to 60.41%, which was significantly higher than that in the other groups after 9 days of aerobic exposure. Furthermore, the abundance of *Lactobacillus* decreased dramatically on the first day of aerobic exposure, and then kept a stable relative abundance in the RB group. However, *Lactobacillus* was still the most abundant bacteria in the SB group during aerobic exposure, and its relative abundance remained above 53.93%. Conspicuous changes were observed that the relative abundance of *Weissella* in the BS group was lower than that in the other groups, while the relative abundance of *Bacillus* in the SB group increased after 9 days of exposure, and was significantly higher than that in the other groups.

### 3.5. Fungal community dynamics in WPCS during aerobic exposure

**Figure 4:**
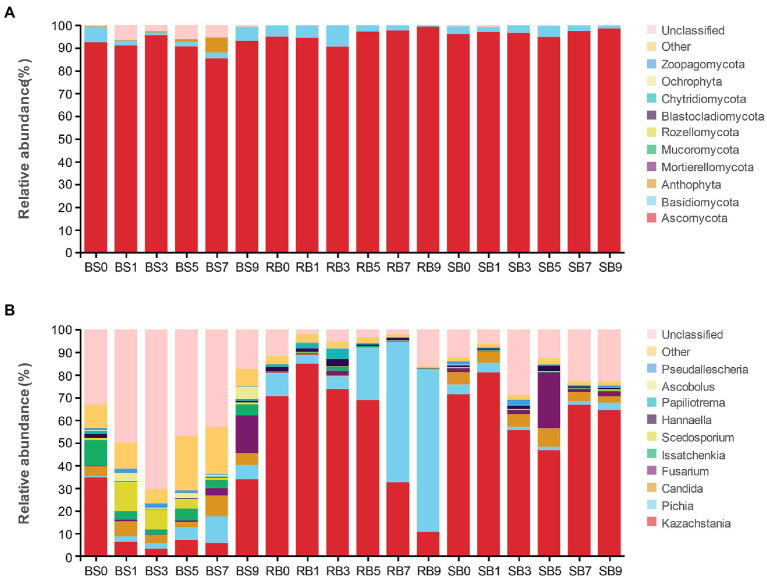
The dynamic fungal community of the WPCS during aerobic exposure. The fungal communities were shown at the phylum level **(A)** and the genus level **(B)**. BS, Bunker silo; RB, Round bale; SB, silage bag. 0, 1, 3, 5, 7, 9: 0, 1, 3, 5, 7, 9 days of aerobic exposure, respectively.

Changes in the fungal community at the phylum level during aerobic exposure are shown in Figure 4A. The phyla Ascomycota, Basidiomycota, Anthophyta, Mortierellomycota, and Mucoromycota were identified in the WPCS. Overall, the predominant ITS detected sequence belonged to the Ascomycota phylum and was followed by lower Basidiomycota, together covering more than 88% of the total sequences observed. Ascomycota was the most abundant phylum in all groups and was also the dominant phylum in the WPCS during the aerobic exposure process.

As shown in Figure 4B, at the genus level, *Kazachstania* and *Pichia* were the dominant genera in the RB group. The abundance of *Kazachstania* in the RB group increased dramatically after 1 day of exposure and then continuously decreased until 9 days. Conversely, the abundance of *Pichia* first decreased within 1 day of aerobic exposure, and then continuously increased until day 9. *Pichia* became the dominant fungus instead of *Kazachstania* after 7 days of exposure. However, neither *Kazachstania* nor *Pichia* showed a remarkable dominance in fungal communities in the BS group from days 1–7. *Fusarium* increased significantly, ranging from 0.64 to 16.72%, from days 5–9 in the BS group. In addition to BS and RB groups, *Kazachstania* was the most abundant fungus in the SB group, followed by *Candida*, *Pichia* and *Fusarium* during the whole aerobic exposure. Moreover, the relative abundance of *Kazachstania* was significantly higher in the SB group than in the other groups from days 7–9. The relative abundance of *Pichia* was observed to be lower in the SB group than in the other groups after aerobic exposure.

### 3.6. Correlation of the bacterial community with fermentation characteristics and the fungal community

**Figure 5:**
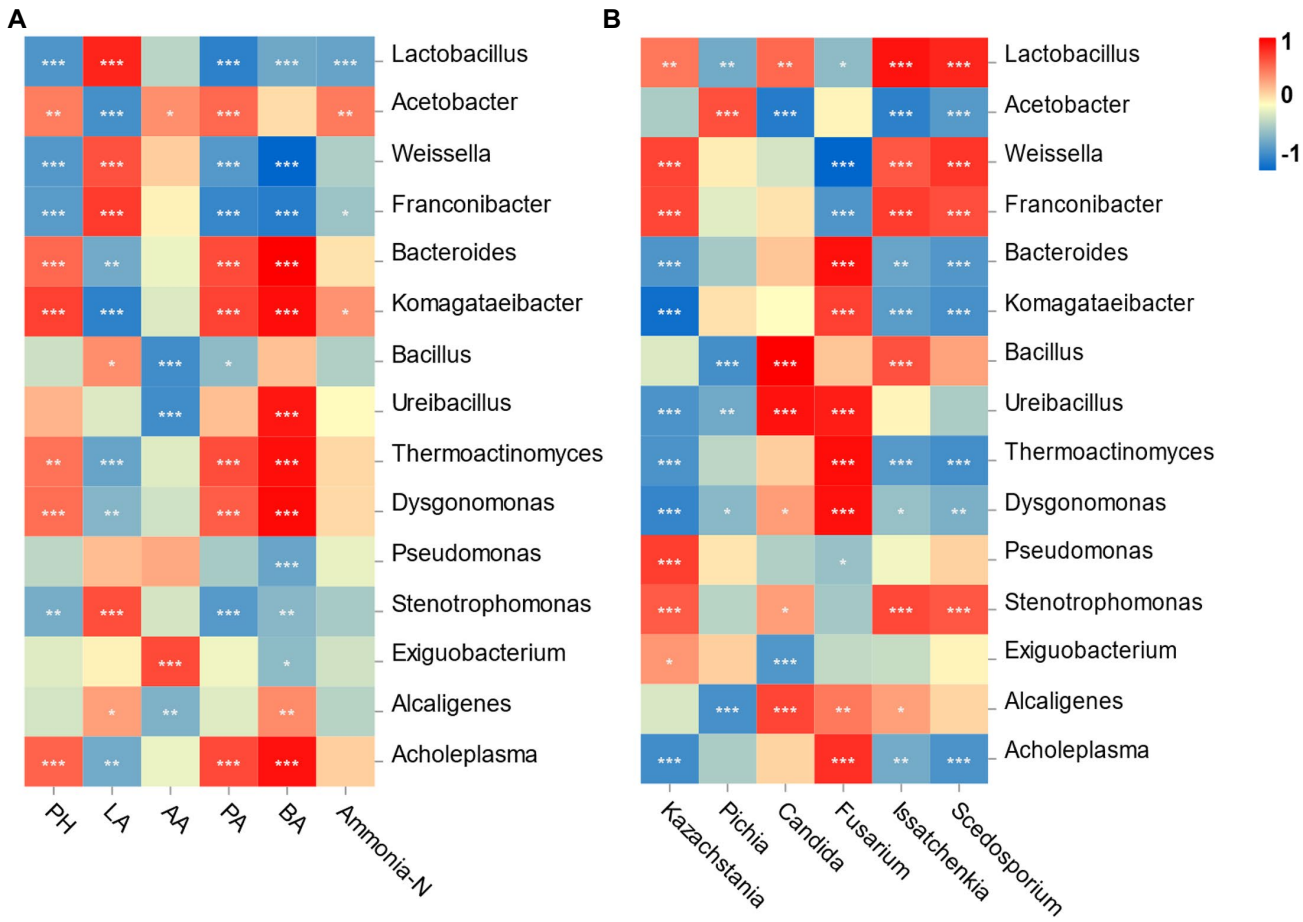
Correlation analysis of the bacterial genus with fermentation characteristic and the top 6 fungi genus. **(A)** *X* and *Y* axis are fermentation factors and bacterial genus, respectively; **(B)** *X* and *Y* axis are genera of fungi and bacteria, respectively. Red squares represent positive correlation (0 < *r* < 1), whereas blue squares represent negative correlation (−1 < *r* < 0). The value of *p* < 0.05 is marked with “*,” *p* < 0.01 is marked with “**,” *p* < 0.001 is marked with “***.”

Spearman’s correlation between the bacterial community and fermentation is shown in Figure 5A. Specifically, the pH value was negatively correlated with the relative abundances of *Lactobacillus* and *Weissella* (*p* < 0.001) and positively correlated with the *Acetobacter* abundance (*p* < 0.01). The LA concentration was significantly positively associated with the abundances of *Lactobacillus* and *Weissella* (*p* < 0.001). The AA content was positively correlated with *Acetobacter* and *Exiguobacterium* abundances (*p* < 0.05) but negatively correlated with *Bacillus* and *Ureibacillus* abundances (*p* < 0.001). The contents of PA and BA were both negatively associated with *Lactobacillus*, *Weissella* and *Franconibater* abundances (*p* < 0.001) and positively correlated with *Bacteroides*, *Thermoactinomyces* and *Dysgonomonas* abundances (*p* < 0.001). Finally, the ammonia-N concentration was negatively correlated with *Lactobacillus* abundances (*p* < 0.001) but positively correlated with *Acetobacter* abundances (*p* < 0.01).

Correlation analysis was performed to illustrate the relationship between bacteria and fungi at the genus level (Figure 5B). *Lactobacillus* and *Weissella* were positively correlated with *Kazachstania* (*p* < 0.01) but negatively correlated with *Fusarium* (*p* < 0.05). *Candida* was positively associated with *Lactobacillus* (*p* < 0.01) but negatively associated with genus *Acetobacter* (*p* < 0.001). *Pichia* was positively correlated with the genera *Acetobacter* (*p* < 0.001) but negatively correlated with *Lactobacillus* (*p* < 0.01).

## 4. Discussion

### 4.1. Effect of different ensiling methods on the fermentation quality, chemical composition and aerobic stability of WPCS after aerobic exposure

Aerobic deterioration is a process that causes nutrient degradation (De Melo et al., 2023). The DM content increased first after aerobic exposure. This was caused by the water and volatile organic compounds in silage volatilized after exposed to the external environment (Merkevičiūte-Venslovė et al., 2023). As expect, the contents of DM, WSC and CP decreased after 9 days of aerobic exposure in this study. DM loss was achieved through the activity of yeasts and molds consuming residual WSC and LA during aerobic exposure (Muck et al., 1991). The higher contents of DM, WSC and LA in the SB group indicated the nutrients were well stored. Limited air exposure due to strong compaction, may illustrate the phenomenon of reduced nutrient degradation in the SB group. Conversely, the contents of ADF and NDF increased during aerobic exposure, which was indirectly caused by the loss of WSC in the WPCS (Randby et al., 2020).

Fermentation characteristics illustrate the fermentation quality of silages (Hao et al., 2022). The pH value of silage is a basic indicator to evaluate microbial activity, and well-fermented WPCS should not exceed a pH of 4.2 (Kung et al., 2018). During 1 days of aerobic exposure, silage was in an unstable state due to the fierce competition between LAB and yeasts. The previous study demonstrated that various organic acids were produced to compete with yeasts and improve the aerobic stability during aerobic exposure (Hu et al., 2020). Hence, the pH value was decreased at 1 day of aerobic exposure. After exposed to the air, the environment changed to aerobic conditions, and oxygen activated the lactate-assimilating yeasts in the WPCS. These yeasts consumed the LA concentration and increased pH value during aerobic exposure (Kung et al., 2003). However, the SB group did not exceed the pH value of 4.2 after 9 days of exposure. This result could be due to the higher LA content and relative abundance of lactic acid bacteria (LAB) observed in SB group than in BS and RB groups (Figure 3B). The higher density of SB may also reduce the entry of oxygen (Tian et al., 2020). Moreover, BA was not observed in the RB and SB groups. The presence of BA may be caused by the metabolic activity of harmful microorganisms, accompanied by substantial DM losses in silages. The contents of ammonia-N in all groups increased with prolonged aerobic exposure. This result is probably due to protein hydrolysis, which is typically caused by extensive protease (Wang et al., 2022). Higher contents of ammonia-N and lower CP were observed in the BS group than in the other groups, illustrating that amino acid deamination was relatively active in BS silages. Overall, with higher contents of LA and lower pH value and PA and ammonia-N contents, the SB group was considered to have lower fermentation degradation after aerobic exposure than the RB and BS groups. In the study, the better aerobic stability of SB group (202 h) was consistent with its lower pH value and higher silage density among all groups. The greater silage density can enhance the aerobic stability of corn silage (Gallo et al., 2018), and the low pH can also improve the aerobic stability to inhibit spoilage microorganisms (Ranjit and Kung, 2000).

### 4.2. Effect of different ensiling methods on mycotoxins levels in WPSC after aerobic exposure

Aflatoxins (AFs) are produced by several species of *Aspergillus* section *Flavi* (Sweeney and Dobson, 1998). AFB1 is considered the most toxic and carcinogenic AF, since it is highly toxic and causes carcinogenic and mutagenic effects in mammals. In the present study, the contents of AFB1 increased with prolonged exposure times, which was similar to the phenomenon reported by Ferrero et al. (2019). Moreover, lower AFB1 contamination was observed in SB than in RB and BS groups. Proper silage management and well-preserved WPCS are essential to inhibit massive concentrations of AFB1. In addition, a higher abundance of LAB (Figure 3B) may also be considered to play a crucial role to limit the accumulation of mycotoxins in SB (Lahtinen et al., 2004).

*Fusarium* toxins are known to be frequently found in corn and animal feed globally (Vaičiulienė et al., 2022). T-2, DON, FB1 and ZEN are secondary metabolites primarily produced by several members of *Fusarium* (Ogunade et al., 2018). The accumulation of oxygen contents allows for spoilage and toxigenic microorganisms to grow continually with prolonged aerobic exposure (Driehuis et al., 2018). Therefore, the concentrations of mycotoxins produced by undesirable microorganisms increased significantly in all groups. Higher *Fusarium* toxin contamination was observed in the BS group than the other groups after 9 days of exposure. This may be caused by the higher relative abundance of *Fusarium* observed in the BS group (Figure 3B). Furthermore, the bunker was prepared on bare ground and thus was highly exposed to the environment (Alonso et al., 2013). A larger exposed area from the ground may cause aerobic microorganism survival and increase the content of mycotoxins in BS silage. Niderkorn et al. (2006) reported that LAB could detoxify *Fusarium* mycotoxins. Thus, the higher abundance of *Lactobacillus* in the SB group than in the other groups may result in reduced accumulation of *Fusarium* mycotoxins. Overall, the contents of AFB1 T-2, ZEN, FB1 and DON in all groups did not exceed the guidance level of adult ruminants after 9 days of exposure (Commission of the European Community, 2006). The silages were opened for exposure from Dec. to Jan., and ambient temperature was detected from 0 to 7.5°C. High temperatures are known to promote aggressive fungal growth (Ogunade et al., 2018). Hence, the lower temperature in the current study may have limited the synthesis of mycotoxins.

### 4.3. Dynamic changes in microbial communities during aerobic exposure

The quality of natural silage depends on the complex composition of the microflora, and the composition of the bacterial community in silage is different at various stages (Yang et al., 2019; Ran et al., 2022). The dominant bacteria at the phylum level were Firmicutes and Proteobacteria in the WPCS during aerobic exposure (Hu et al., 2018), which was supported by the present study. Proteobacteria became the dominant phylum instead of Firmicutes in the BS and RB groups after 1 day of exposure. This result indicated that the WPCS involved a notable shift in the bacterial community from Firmicutes to Proteobacteria after the environment changed from anaerobic to aerobic (Liu et al., 2019). At the genus level, *Acetobacter* showed a predominance of the BS and RB groups during oxygen exposure. *Acetobacter* are often regarded as an initiation of aerobic deterioration, which causes silage deterioration and further limits *Lactobacillus* proliferation (Guan et al., 2018). A high abundance of *Acetobacter* is usually found in bunker silages (Wang et al., 2014), which was observed in the current study. However, *Lactobacillus* still dominated the structure of the bacterial community in the SB group, although the abundance of *Lactobacillus* decreased after 9 days of oxygen exposure. *Lactobacillus* is well known as a LAB in maize silages and has been reported by many studies (Guan et al., 2018; Hu et al., 2018; Huang et al., 2021). *Lactobacillus* could inhibit fungal growth, and prevent them from becoming dominant microorganisms in the early stage of aerobic exposure (Guan et al., 2020). In addition, *Lactobacillus* has an excellent ability to remove mycotoxins (Hathout and Aly, 2014). A higher relative abundance of *Bacillus* was also detected in the SB group during aerobic exposure. Some *Bacillus* species can produce bacteriocin to inhibit the growth of some undesirable microbes (Lara et al., 2016). Moreover, these bacteria have also been found to be effective in the removal of mycotoxins from liquid medium (Hathout and Aly, 2014). Therefore, the lower mycotoxin level and undesirable microbes in the SB group than in the other groups may be caused by the higher relative abundances of *Bacillus* and *Lactobacillus*.

Due to the response to oxygen exposure, various fungi began to compete with the dominance of the microbial community in silages. The majority of fungi in corn silage belonged to Ascomycota, followed by Basidiomycota (May et al., 2001). At the genus level, *Kazachstania*, *Pichia* and *Candida* were the top 3 most abundant fungi in the WPCS (Xu et al., 2019). *Kazachstania*, *Pichia*, and *Candida* belong to the Saccharomycetes, regarded as yeasts frequently through culture-based methods (Wang et al., 2020). It is well known that *Pichia* species are often considered the major initiators of silage aerobic deterioration. The predominant fungal genus shifted to *Pichia* in the RB group after aerobic exposure, illustrating that the composition of the fungal community was significantly changed during oxygen exposure. *Kazachstania* is usually observed as the dominate genus in WPCS after aerobic exposure and is associated with the deterioration of silage (Dolci et al., 2011; Xu et al., 2019). *Candida* can assimilate LA and promote WPCS deterioration (Pahlow et al., 2003). To respond to oxygen exposure, yeasts became active and utilized various organic acids, resulting in a continual increase in pH value. Thus, the proliferation of massive spoilage microorganisms in silages was promoted, leading to silage deterioration and mycotoxin accumulation (McAllister et al., 1995). It is also worth noting that *Fusarium* appeared in silages during aerobic exposure. This result may also cause mycotoxin accumulation, as shown in Table 3.

### 4.4. Correlation analysis of the bacterial community with fermentation characteristics and the fungal community

Silage quality was influenced by the bacterial community through a series of metabolites. The *Lactobacillus* abundance had a positive correlation with the concentration of LA (Huang et al., 2021), and *Weissella* and *Lactobacillus* abundance showed a negative correlation with the pH value (Yang et al., 2019). The results of these studies were supported by the current research. *Acetobacter* can consume ethanol to produce AA after exposure to air (Nanda et al., 2001). This can be proven by the content of AA being positively correlated with *Acetobacter*, and higher contents of AA were found in the RB group. The positive correlation between *Acetobacter* and ammonia-N contents illustrated that the existence of *Acetobacter* probably caused the degradation of protein in this study.

It is also important to explore the relationship between bacteria and fungi to understand the decomposition of WPCS. Yeasts play a vital role in facilitating the symbiosis of various microorganisms (Alvarez-Martin et al., 2008). After aerobic exposure, there was a synergistic effect between yeasts and LAB (Roostita and Fleet, 1996). *Kazachstania* and *Candida* can metabolize organic acids, and metabolites associated with nutrients help LAB grow. In addition, LAB could also promote a suitable environment for *Kazachstania* and *Candida* to multiply after aerobic exposure (Wang et al., 2020). The accumulation of *Fusarium*-derived mycotoxins is caused by the metabolic activity of toxigenic strains of *Fusarium* genus, and AFB 1 is mainly produced by toxigenic *Aspergillus* species (Kalúzová et al., 2022). However, the direct relationship between the *Aspergillus* and AFB 1 in WPCS was not found. Similarly, the previous study also reported that no correlations were observed between fungal DNA and mycotoxin contents (Vandicke et al., 2021). A negative correlation of *Fusarium* with *Lactobacillus* and *Weissella* was found in this study. It is known that *Fusarium* cannot tolerate a low pH environment (Cheli et al., 2013). Therefore, the higher relative abundance of *Lactobacillus* and lower pH in the SB group than in the other groups may have inhibited the proliferation of *Fusarium* and further limited the accumulation of *Fusarium* mycotoxins.

## 5. Conclusion

This study analyzed the effects of different ensiling methods on nutritional characteristics, fermentation quality, aerobic stability, mycotoxin levels and microbial communities in whole-plant corn silage after aerobic exposure. The WPCS in SB group deteriorated later than other groups, and the losses of DM, CP and WSC contents in SB silage were also less serious. A considerably lower proportion of mycotoxins was observed in SB than in BS groups after 9 days of aerobic exposure, indicating the higher safety of SB to ruminants. The fungal community was affected by different ensiling methods. After 9 days of aerobic exposure, *Pichia* was the predominant fungal genus in the RB group, while *Kazachstania* was the most abundant fungus in the SB group. Although exposed to air, *Lactobacillus* still dominated the bacterial community of SB and reduced fermentation degradation, as shown by a higher level of LA and a lower level of BA, ammonia-N and pH value. Therefore, with a lower pH value and the dominance of *Lactobacillus* in SB, the proliferation of toxic microorganisms was limited.

## Data availability statement

The original contributions presented in the study are included in the article/supplementary material, further inquiries can be directed to the corresponding author.

## Author contributions

G-hX, JH, and M-zZ designed the experiment. G-hX, YH, and C-rW performed the experiment, analysis, and writing. FY, H-yY, CC, and JH performed the editing and revision. All authors have read and agreed to the published version of the manuscript.

## Funding

This research was supported by Scientific Research Cultivation Project of Guizhou University [(2020)17], Major Special Project of Science and Technology of Guizhou Province [(2020)3009-2], Science and Technology Support project of Guizhou Province [(2021)-043], and Guizhou Talent Base of Grassland Ecological Animal Husbandry (RCJD2018-13).

## Conflict of interest

The authors declare that the research was conducted in the absence of any commercial or financial relationships that could be construed as a potential conflict of interest.

## Publisher’s note

All claims expressed in this article are solely those of the authors and do not necessarily represent those of their affiliated organizations, or those of the publisher, the editors and the reviewers. Any product that may be evaluated in this article, or claim that may be made by its manufacturer, is not guaranteed or endorsed by the publisher.
